# Predicting pathologic complete response in locally advanced rectal cancer patients after neoadjuvant therapy: a machine learning model using XGBoost

**DOI:** 10.1007/s00384-022-04157-z

**Published:** 2022-06-15

**Authors:** Xijie Chen, Wenhui Wang, Junguo Chen, Liang Xu, Xiaosheng He, Ping Lan, Jiancong Hu, Lei Lian

**Affiliations:** 1 Guangdong Provincial Key Laboratory of Colorectal and Pelvic Floor Diseases, Guangdong Institute of Gastroenterology, The Sixth Affiliated Hospital, Sun Yat-Sen University, Guangzhou, Guangdong China; 2 Department of Gastrointestinal Surgery, The Sixth Affiliated Hospital, Sun Yat-Sen University, Guangzhou, Guangdong China; 3 Department of Colorectal Surgery, The Sixth Affiliated Hospital, Sun Yat-Sen University, Guangzhou, Guangdong China; 4 Department of Pathology, The Sixth Affiliated Hospital, Sun Yat-Sen University, Guangzhou, Guangdong China; 5 Department of Endoscopic Surgery, The Sixth Affiliated Hospital, Sun Yat-Sen University, Guangzhou, Guangdong China; 6 Department of Network Management, The Sixth Affiliated Hospital, Sun Yat-Sen University, Guangzhou, Guangdong China; 7 Information and Data Centre, Guangzhou First People’s Hospital, School of Medicine, South China University of Technology, Guangdong Guangzhou, China

**Keywords:** Machine learning, Rectal cancer, Neoadjuvant therapy, Complete response

## Abstract

**Purpose:**

Watch and wait strategy is a safe and effective alternative to surgery in patients with locally advanced rectal cancer (LARC) who have achieved pathological complete response (pCR) after neoadjuvant therapy (NAT); present restaging methods do not meet clinical needs. This study aimed to construct a machine learning (ML) model to predict pCR preoperatively.

**Methods:**

LARC patients who received NAT were included to generate an extreme gradient boosting-based ML model to predict pCR. The group was divided into a training set and a tuning set at a 7:3 ratio. The SHapley Additive exPlanations value was used to quantify feature importance. The ML model was compared with a nomogram model developed using independent risk factors identified by conventional multivariate logistic regression analysis.

**Results:**

Compared with the nomogram model, our ML model improved the area under the receiver operating characteristics from 0.72 to 0.95, sensitivity from 43 to 82.2%, and specificity from 87.1 to 91.6% in the training set, the same trend applied to the tuning set. Neoadjuvant radiotherapy, preoperative carbohydrate antigen 125 (CA125), CA199, carcinoembryonic antigen level, and depth of tumor invasion were significant in predicting pCR in both models.

**Conclusion:**

Our ML model is a potential alternative to the existing assessment tools to conduct triage treatment for patients and provides reference for clinicians in tailoring individual treatment: the watch and wait strategy is used to avoid surgical trauma in pCR patients, and non-pCR patients receive surgical treatment to avoid missing the optimal operation time window.

**Supplementary Information:**

The online version contains supplementary material available at 10.1007/s00384-022-04157-z.

## Introduction

Neoadjuvant therapy (NAT) followed by total mesorectal excision (TME) is the standard of care for locally advanced rectal cancer (LARC) with curative intent. NAT was shown to downsize and downstage cancer, increase the R0 resection rate, and thus improve oncological outcomes [1, 2]. Treatment response to NAT varies between individuals, from insensitive to pathologic complete response (pCR), with no visible cancer cells in the pathologic specimens, accounting for 20% of the population [3]. The prognosis of pCR patients is better than that of non-pCR patients. Nowadays, preoperative tumor restaging after NAT is generally studied by digital rectal examination, endoscopic ultrasonography, magnetic resonance imaging (MRI), and endoscopy in clinical practice. For those who attain clinical complete response (cCR, predict as pCR preoperative), an organ-sparing protocol called watch and wait (W&W) strategy has received considerable attention in recent years. This strategy refers to the option to exempt patients from major surgery and implement intensive surveillance in the first 3 years after NAT [4]. Previous studies have demonstrated the safety and effectiveness of W&W in cCR patients, yielding little oncologic risk other than the risk of surgery [4–11]. However, the gold standard of tumor response is only available in surgical specimens. Definition of cCR lacks uniform criteria because human assessment introduce subjectivity; as a result, the reported cCR rates range from 10 to 78%, followed by tumor regrowth rates ranging from 7 to 33% [9, 12–14]. For some examples,  a prospective cohort study comprising 385 LARC patients with cCR revealed that 87 patients developed local recurrence during the near 3-year follow-up period, of whom 97% could receive curative resection, and uncontrolled pelvic disease was 1.6% [15]. Data from the International Watch & Wait Database, which has 47 participating institutions from 15 countries in Europe, revealed a 2-year accumulated local regrowth rate of 25% in cCR patients, and 97% of them were located in the bowel wall, which requires salvage surgery [9].

With the improvement of survival outcome, patients pay more attention to pursue a higher quality of life (QOL). Nonoperative management in a highly selected population was proven to be feasible as it spares permanent colostomy, sphincterotomy, bladder, and sexual dysfunction, and avoids 1–5% surgical mortality and 34–39% morbidity, thus ensuring good QOL [16–19]. Therefore, for restaging unmet clinical needs, a more precise and easy-to-use tool with high performance on pCR prediction is imperative.

Machine learning (ML), a branch of artificial intelligence and computer science, focusing on simulating the way the human brain learns using statistics and algorithms to improve accuracy, has had an outstanding performance in disease diagnosis, prognosis prediction, antitumor drug response, and treatment response assessment [20–25]. However, studies evaluating tumor response in LARC after NAT using ML algorithms are limited. In this study, we aimed to integrate some commonly used preoperative clinicopathological parameters to develop an ML classifier to predict tumor response of LARC after NAT, and compare its performance with nomogram constructed by the conventional logistic regression method, with the aim to provide reference for individualized treatment.

## Methods

### Ethical statement

This study was approved by the Institutional Review Board of the Sixth Affiliated Hospital, Sun Yat-sen University (No. 2021ZSLYEC-063), and complied with the Declaration of Helsinki (World Medical Association Declaration of Helsinki 2013). All the patients gave informed consent to allow their electronic medical records to be used for cancer research voluntarily during their first visit to the hospital, and data were desensitized at the beginning of statistical analyses to protect patients’ privacy.

### Study population

We included patients diagnosed with rectal cancer from 2010 to 2020 at the Sixth Affiliated Hospital, Sun Yat-sen University. Patient lists were extracted from pathology report records, and only those who received NAT were selected. Neoadjuvant chemotherapy was based on oral/intravenous 5-fluorouracil or combined with oxaliplatin/irinotecan. The neoadjuvant radiotherapy regimen was a long-term radiotherapy of 50 Gy in 25 fractions, or a short-term radiotherapy regimen of 5 Gy per day for 5 consecutive days.

The eligibility criteria were as follows: (1) T3–4/N + (conventionally staged by MRI and computed tomography), (2) age 18–75 years, (3) received a full course of NAT, (4) regular restaging that adhered to the treatment protocol, (5) underwent radical surgery, and (6) tumor regression grade information available in the surgical pathology report. Patients were excluded if they had (1) multiple synchronous tumors or newly discovered tumors during treatment; (2) familial adenomatous polyposis, inflammatory bowel disease, or other diseases that predispose to colorectal cancer; and (3) local excision of the primary tumor only.

The patients were divided into a training set and a tuning set (similar to internal validation set) at a 7:3 ratio. The training set was used to build binary classifiers, which in turn was used to predict the cluster labels on the tuning set (held-out samples).

### Clinicopathological selection and outcome definition

Patient, tumor, and treatment information were extracted from the electronic medical records. Patient information mainly included demographic characteristics (sex, age, height, weight, body mass index [BMI], family history, and tumor history); tumor information included radiology findings (MRI: tumor invasion depth, distance from the anal verge, tumor location, tumor size, and tumor recurrence), endoscopic findings (tumor size by endoscopy: the proportion that the tumor occupied the intestinal lumen), and peri-NAT serological tumor biomarkers (cancer antigen 125 [CA125], CA199, CA153, carcinoembryonic antigen [CEA], and alpha-fetoprotein [AFP]). T1, T2, and DWI sequences were used to comprehensively translate tumor information on MRI. Given that CEA and CA199 play important roles in colorectal cancer progression and recurrence surveillance, we postulated that the changes in these biomarkers may reflect the microscopic alterations in the tumor after NAT; hence, we included the CEA difference, ratio of CEA difference, CA199 difference, and ratio of CA199 difference into the analysis. Treatment information included neoadjuvant radiotherapy and neoadjuvant chemotherapy regimen. The oncological outcome was pCR, which was determined by tumor regression grade 0 (TRG0) without lymph node metastasis; otherwise, it was non-pCR (TRG1-3). TRG was justified in accordance with the National Comprehensive Cancer Network guidelines [26]. In this study, the area under the receiver operating characteristics (AUROC) was considered the primary index to evaluate the performance of the models, with sensitivity and specificity as secondary evaluation indexes.

### Model development

#### ML framework

An ML ensemble algorithm based on extreme gradient boosting (XGBoost) [27] was used to predict tumor response in LARC patients. XGBoost easily captures the nonlinear relationships between features and classification labels based on the concept of “exact greedy algorithm.” Essentially, it aggregates multiple weak classifiers with limited accuracy into stronger ones by establishing a series of classification and regression trees (CARTS) iteratively with a highly adaptive approach. This was performed by iteratively fitting decision trees, with each iteration targeting the prediction residuals (also known as the loss function) of the preceding tree. The XGBoost algorithm optimizes the loss function via second-order Taylor expansion; meanwhile, a regularization term that contained leaf nodes was applied to control the model complexity to avoid over-fitting. Gains or losses after splitting of previous lead nodes determined further splitting, which was repeated until the minimum loss function and complexity of the model was less than the threshold setting. Therefore, the final predicted probability was calculated for all trees.

When training models, hyperparameters, including max_depth, subsample, and colsample_by tree, were optimized separately for each tree in the training set by random sampling. In addition, the effect of imbalanced data was corrected by oversampling and undersampling [28, 29], and the XGBoost model was trained with the generated effectively balanced dataset. To further strengthen generalizability, a five-fold cross validation was used to split the whole training set into five subsets, with four subsets as the training set and the rest as the test subset each time until each subset was tested. For the features, the average value of the five trained XGBoost models was taken to measure their importance. SHapley Additive exPlanations (SHAP) [30] value was calculated to interpret the marginal contribution of the model features and explain the output of the model.

#### Nomogram model building

To compare ML models with conventional linear models, univariate analyses were used to identify factors that correlated with tumor response and incorporate them into a binary stepwise multivariate logistic regression model to identify independent risk factors. Thereafter, we constructed a nomogram model that used these independent risk factors and validated them internally. Harrell’s concordance index (C-index) and AUROC were calculated to examine the predicted power of the nomogram, and a calibration curve was drawn to judge the discrepancy between predicted and actual events.

### Statistical analyses

Data are presented as mean ± standard deviation for continuous variables with normal distribution, medians with interquartile ranges for continuous variables with non-normal distribution, and frequency (percentage) for categorical variables. During nomogram construction, some continuous variables were transformed into categorical variables for analysis. *t*-Test/Mann–Whitney test or chi-square were used to conduct univariate analysis where appropriate, and multivariate analyses were implemented using the logistic regression method. The variables significantly related to pCR in univariate analysis were included in multivariate regression analysis, and the independent risk factors obtained from multivariate regression analysis were used for nomogram modeling. All statistical analyses were conducted using SPSS software (version 26.0 for Windows, IBM Corp., Armonk, NY, USA) and R software (version 3.4.1, The R Foundation for Statistical Computing, Austria). The ML model was developed by the “xgboost” package, “shapforxgboost” package, and “imbalance” package. ROC was plotted by the “pROC” package. A two-sided *p* value < 0.05 was considered significant.

## Results

### Patient characteristics

**Fig. 1:**
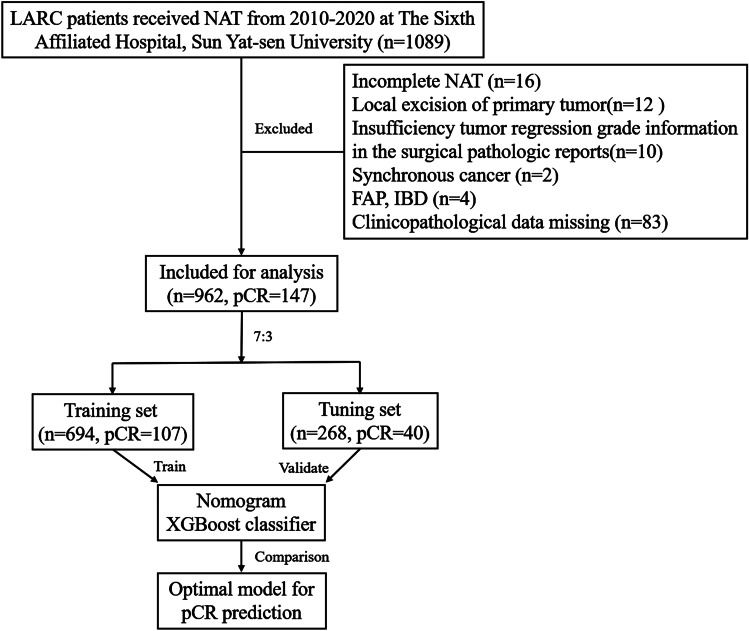
Flow chart of the study design

A total of 962 consecutive LARC patients during the 2010–2020 period from the Sixth Affiliated Hospital, Sun Yat-sen University, were retrospectively included in this study (Fig. 1). The training and tuning sets included 694 and 268 patients, respectively, with a comparable pCR rate between the groups (15.4% vs 14.9%). Compared with the training set, clinical T stage evaluated qualitatively by the depth of tumor invasion was more advanced with 100% T3–4 tumors. A higher prevalence of upper-third rectal cancer, a higher level of preoperative CEA, and a tendency of larger tumor size by colonoscopy were observed, which combined in all manifestations of more invasive tumors in the tuning set. In addition, the proportion of radiotherapy was lower, and chemotherapy regimens were significantly different from the training set, which may help explain the poorer downward trend in CEA difference and ratio of CEA difference for LARC patients after receiving NAT in the tuning set (Table 1). Other clinicopathological parameters were comparable between groups.

**Table 1:** Demographic characteristics of LARC patients who received neoadjuvant therapy

Variables	All	Training set	Tuning set	*p* value
Gender				0.936
Man	691 (71.8%)	499 (71.9%)	192 (71.6%)	
Woman	271 (28.2%)	195 (28.1%)	76 (28.4%)	
Age	57 (47.0, 64.0)	56 (46, 64)	57 (48.0, 65.0)	0.211
BMI				0.098
< 18.5	83 (8.6%)	64 (9.2%)	19 (7.1%)	
18.5 ≤ X < 24	575 (59.8%)	424 (61.1%)	151 (56.3%)	
≥ 24	304 (31.6%)	206 (29.7%)	98 (36.6%)	
Family history of cancer				0.178
Yes	15 (1.6%)	8 (1.2%)	7 (2.6%)	
No	947 (98.4%)	686 (98.8%)	261 (97.4%)	
History of cancer				0.665
Yes	3 (0.3%)	3 (0.4%)	0 (0%)	
No	959 (99.7%)	691 (99.65%)	268 (100%)	
Differentiation				0.902
Well	260 (27.0%)	189 (27.2%)	71 (26.5%)	
Medium	605 (63.0%)	438 (63.1%)	167 (62.3%)	
Poorly	63 (6.5%)	43 (6.2%)	20 (7.5%)	
Undifferentiation	34 (3.5%)	24 (3.5%)	10 (3.7%)	
Depth of tumor invasion				< 0.0001
1–2	72 (7.5%)	72 (10.4%)	0 (0)	
3–4	890 (92.5%)	622 (89.6%)	268 (100%)	
Distance of tumor from the anal	5.5 (3.9, 7.6)	5.4 (3.8, 7.5)	6.0 (4.0, 8.0)	0.062
tumor location				< 0.0001
Upper	71 (7.4%)	37 (5.3%)	34 (12.7%)	
Middle	439 (45.6%)	334 (48.1%)	105 (39.2%)	
Lower	452 (47%)	323 (46.6%)	129 (48.1%)	
Tumor size by MRI	3.0 (2.2, 4.0)	3.0 (4.0, 2.2)	3.0 (2.3, 3.7)	0.19
Recurrent tumor				1
Yes	9 (0.9%)	6 (0.9%)	3 (1.1%)	
No	953 (99.1%)	688 (99.1%)	265 (98.9%)	
Tumor size by colonoscopy	0.5 (0.33, 1.0)	0.5 (0.33, 0.81)	0.5 (0.4, 1.0)	0.069
Initial CA125	9.4 (6.6, 13.0)	9.2 (6.5, 12.9)	9.8 (6.63, 13.2)	0.526
Initial CEA	4.1 (2.1, 9.9)	4.0 (2.1, 9.1)	4.8 (2.2, 10.5)	0.157
Initial CA199	9.2 (4.2, 21.9)	9.2 (4.1, 21.3)	9.2 (4.4, 23.1)	0.449
Initial CA153	7.5 (5.8, 11.3)	7.5 (5.5, 11.1)	7.4 (5.7, 11.5)	0.626
Initial AFP	2.6 (2.0, 3.7)	2.6 (1.9, 3.7)	2.6 (2.0, 3.7)	0.984
Preoperative CA125	10.6 (7.6, 14.5)	10.4 (7.4, 14.6)	10.8 (7.7, 14.2)	0.736
Preoperative CEA	2.9 (1.7, 5.0)	2.5 (1.6, 4.5)	3.5 (2.2, 6.0)	< 0.0001
Preoperative CA199	7.3 (3.5, 15.5)	7.5 (3.5, 15.2)	7.2 (3.6, 16.2)	0.660
Preoperative CA153	10.2 (7.5, 15.0)	10.2 (7.5, 15.0)	10.3 (7.6, 15.1)	0.583
Preoperative AFP	3.4 (2.5, 4.9)	3.5 (2.5, 4.9)	3.3 (2.5, 4.8)	0.497
CEA difference	−0.8 (−5.1, 0.3)	−0.9 (−5.1, 0.1)	−0.4 (−4.9, 1.1)	0.001
Ratio of CEA difference	−0.3 (−6.2, 0.1)	−0.3 (−0.6, 0.1)	−0.1 (−0.6, 0.4)	< 0.0001
CA199 difference	−0.3 (−0.6, 0.1)	−0.3 (−6.7, 1.4)	−0.2 (−7.3, 1.2)	0.802
Ratio of CA199 difference	−0.1 (−0.5, 0.2)	−0.1 (−0.5, 0.2)	−0.1 (−0.5, 0.2)	0.847
Radiotherapy				< 0.0001
Yes	442 (45.9%)	358 (51.6%)	84 (31.3%)	
No	520 (54.1%)	336 (48.4%)	184 (68.7%)	
Chemotherapy				< 0.0001
Single-agent	145 (15.1%)	119 (17.1%)	26 (9.7%)	
Double-agent	651 (67.7%)	462 (66.6%)	189 (70.5%)	
Triple-agent	159 (16.5%)	113 (16.3%)	46 (17.2%)	
Unknown	7 (0.7%)	0 (0)	7 (2.6%)	
pCR				0.849
Yes	147 (15.3%)	107 (15.4%)	40 (14.9%)	
No	815 (84.7%)	587 (84.6%)	228 (85.1%)	

*LARC*, locally advanced rectal cancer; *BMI*, body mass index; *pCR*, pathologic complete response; *CA199*, carbohydrate antigen 199; *CA125*, carbohydrate antigen 125; *AFP*, alpha-fetoprotein; *CEA*, carcinoembryonic antigen

### ML model building and validation

**Fig. 2:**
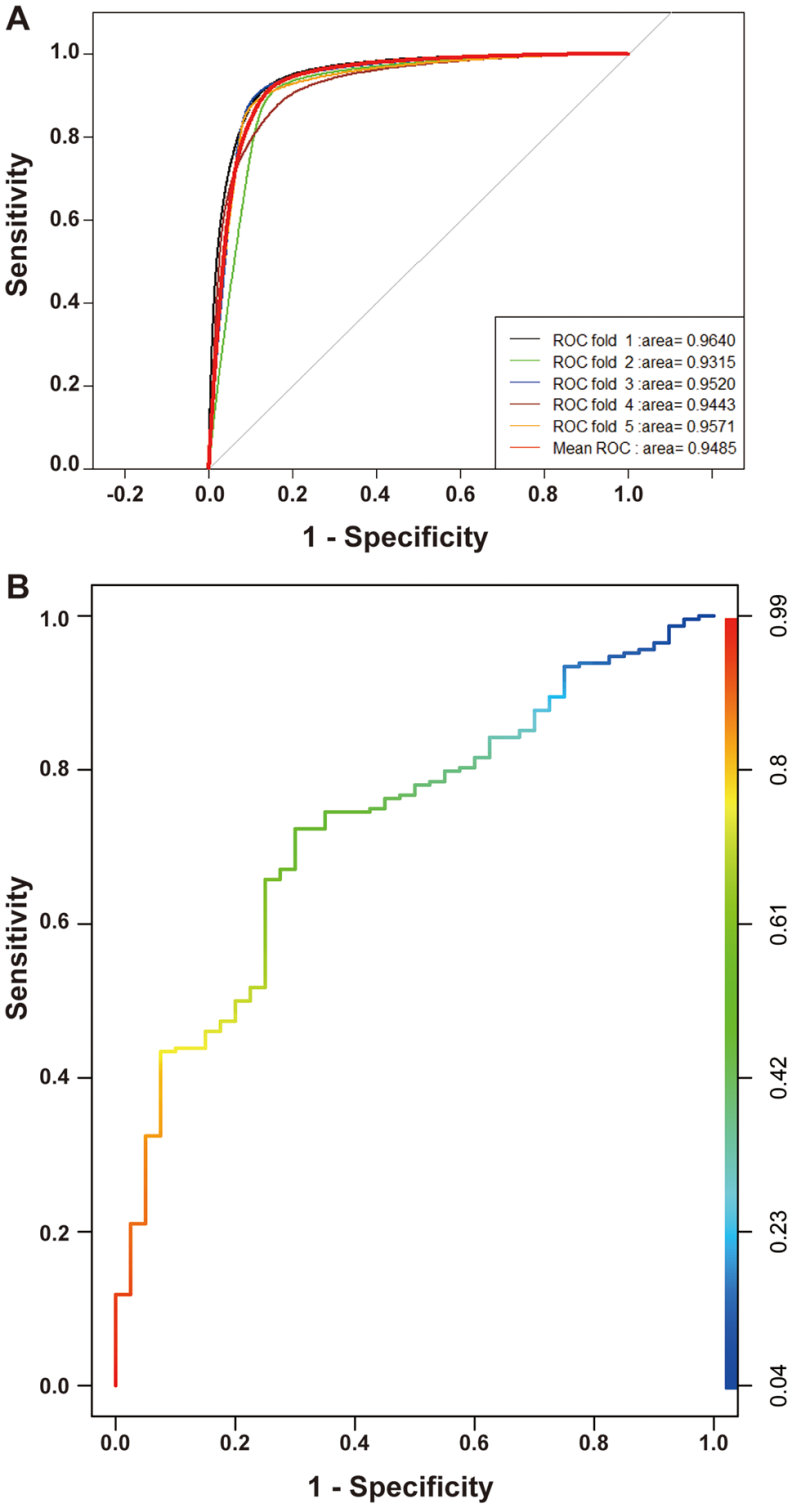
ROC curve for assessing clinical performance of the ML model. **A** ROC curve generated by five-fold cross validation in the training set. **B** ROC curve in the tuning set. ROC, receiver operating characteristics curve; ML, machine learning

**Fig. 3:**
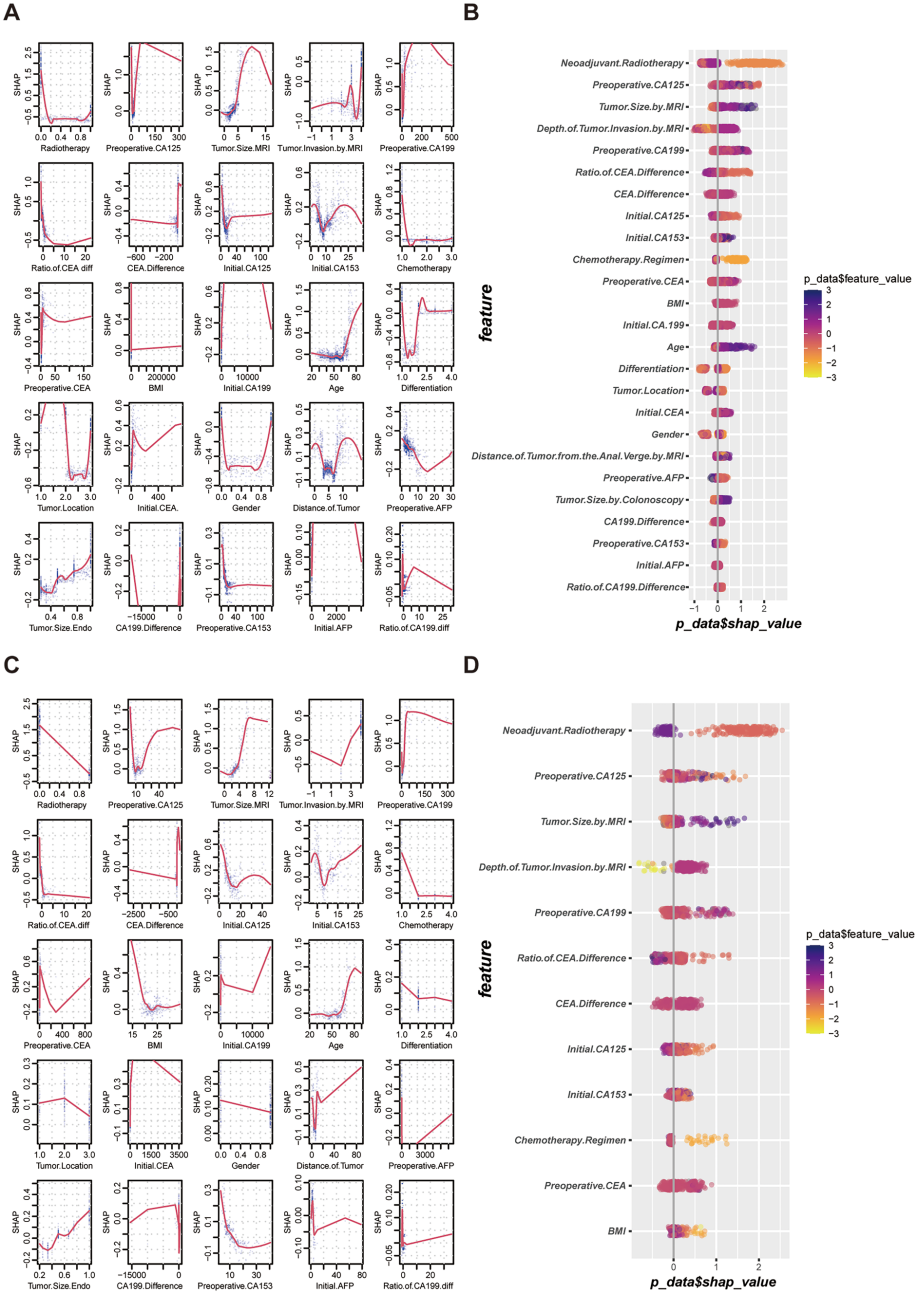
SHAP value distribution of each sample in different variables and feature importance rankings to predict outcomes of the model. **A** Nonlinear distribution of each feature in the training set: the higher the absolute value of SHAP, the stronger the effect on the outcomes. **B** Feature importance rankings in the training set. The horizontal axis represents the relationship between each feature and the probability of pCR. The longitudinal axis shows the variable names. Feature importance rankings in a descending order are dependent on the average values of SHAP. The color indicates the SHAP value of the feature where high value is coded in dark purple (positive impact) and dark yellow (negative impact) and a low value is coded in light purple and light yellow: the darker the color, the stronger the prediction. **C** Nonlinear distribution of each feature in the tuning set. **D** Feature importance rankings in the tuning set. pCR, pathological complete response; SHAP, SHapley Additive exPlanations

Twenty-eight variables were used as inputs to determine their nonlinear relationships with category labels. We first processed the training set to ensure a balanced and representative dataset, then started running the ML model with default hyperparameters, and adjusted them into an optimal model step by step. Finally, we stopped training at “max_depth” of 10, “eta” of 0.2, “gamma” of 0.9, and “scale_pos_weight” of 0.1 after repeated attempts. As displayed in Fig. 2, the AUROC of the XGBoost model was 0.95, with a sensitivity of 82.2% and specificity of 91.6%, which indicated a perfect fit between the predicted results and actual events. In addition, we used the SHAP value to quantify feature importance, calculated feature importance scores (Fig. 3A) for each sample, and averaged features to obtain their final importance ranks (Fig. 3B). As shown in Fig. 3A, we were aware of the nonlinear relationship between variables and labels that conventional statistical methods could ignore. Figure 3B shows that neoadjuvant radiotherapy, preoperative CA125, tumor size by MRI, and depth of tumor invasion by MRI ranked the top four in terms of importance in our ML model, while CA199 difference, preoperative CA153, initial AFP, and ratio of CA199 difference made little contribution. Unsurprisingly, the results in the tuning set were highly consistent with the training set, with AUROC, sensitivity, and specificity of 0.73, 71.93%, and 70%, respectively. For the feature importance evaluation, both sets showed similar trends.

### Nomogram construction and validation

**Fig. 4:**
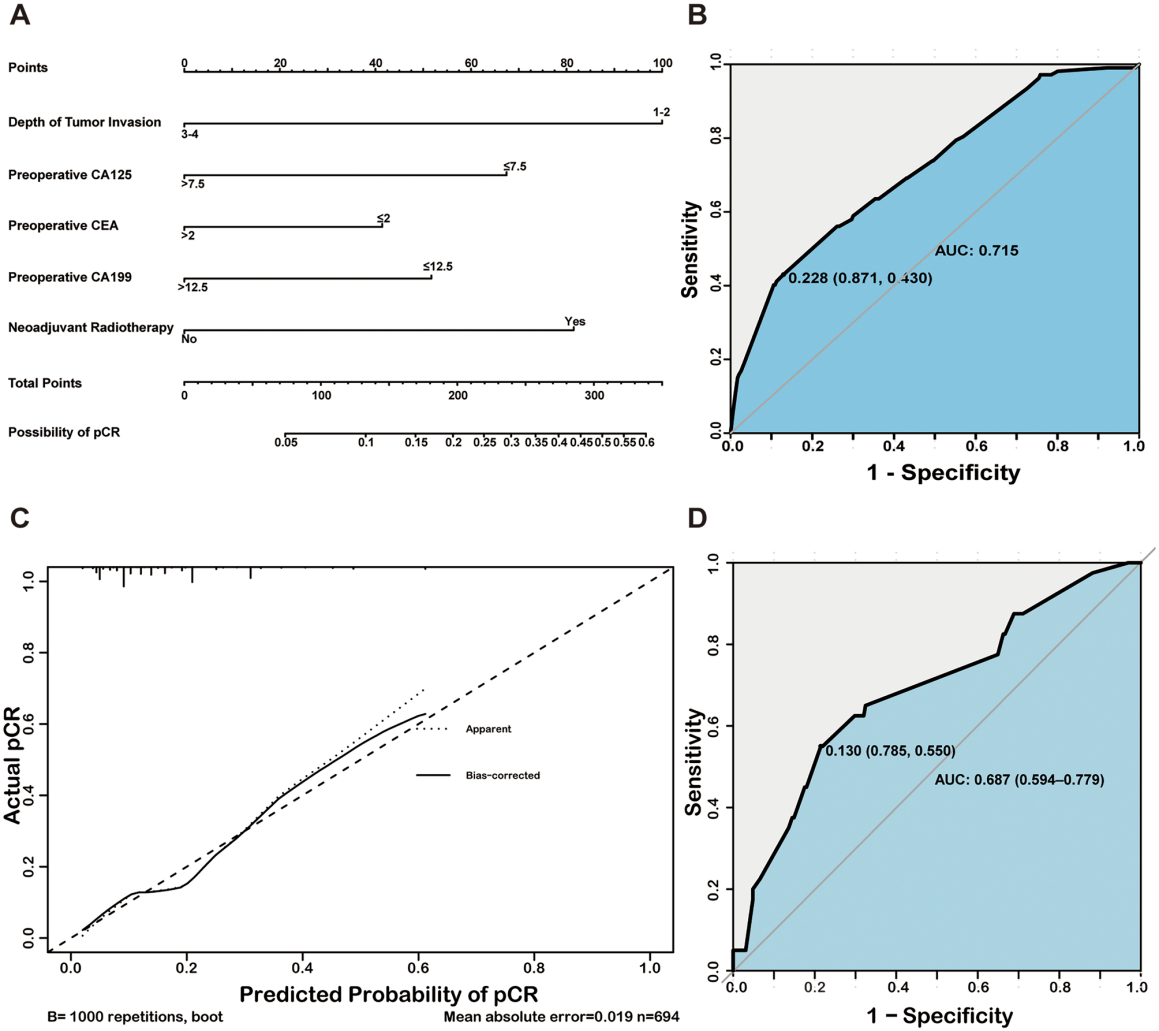
Nomogram construction and validation in both training and tuning sets. **A** The total points are calculated by adding the point value of each variable, which is decided by drawing a straight line up to the point axis. The probability of pCR is determined by drawing a straight line down from the total point axis. **B** ROC curve to evaluate the performance for predicting pCR in the training set. **C** The calibration curve of the training set shows the fitness of the predictive events to the actual events. The 45° dotted lines represent the ideal status with a 100% accuracy. The apparent line represents the predictive ability of the model; the closer the apparent line to the ideal line, the more precise is the model. **D** ROC curve to evaluate the performance for predicting pCR in the tuning set. ROC, receiver operating characteristics; pCR, pathologic complete response

Univariate analysis revealed that the depth of tumor invasion, tumor size on MRI, tumor size by colonoscopy, initial CEA level, preoperative CA125 level, preoperative CEA, preoperative CA199, and neoadjuvant radiotherapy were significantly associated with tumor response. Then, we incorporated these factors into a multivariate stepwise regression analysis to find preoperative CA125, preoperative CEA, preoperative CA199, and neoadjuvant radiotherapy were independent risk factors (Table 2). A nomogram model was then applied to quantitatively visualize the contribution of each variable based on the multivariate analysis (Fig. 4A). As a result, the C-index (equal to AUROC) was 0.72, indicating a moderate predictive ability (Fig. 4B). Calibration showed that the predictive curve was close to the ideal curve (Fig. 4C). Using optimal threshold criteria, this model can accurately distinguish pCR from non-pCR with 43% sensitivity and 87.1% specificity. A similar result was obtained in the validation group (AUROC, 0.69; sensitivity, 55%; specificity, 78.5%) (Fig. 4D).

**Table 2:** Univariate and multivariate analyses for identifying risk factors associated with binary tumor response in LARC patients who received neoadjuvant therapy

Variables	Univariate analysis			Multivariate analysis	
	Non-pCR	pCR	*p* value	OR (95%CI)	*p* value
Gender			0.492		
Man	425 (72.4%)	74 (69.2%)			
Woman	162 (27.6%)	33 (30.8%)			
Age			0.351		
< 50	187 (31.9%)	39 (36.4%)			
≥ 50	400 (68.1%)	68 (63.6%)			
BMI			0.209		
< 18.5	59 (7.7%)	5 (4.7%)			
18.5 ≤ X < 24	355 (62.8%)	69 (64.5%)			
≥ 24	173 (29.5%)	33 (30.8%)			
Family history of cancer			0.470		
Yes	8 (1.4%)	0 (0)			
No	579 (98.6%)	107 (100%)			
History of cancer			1		
Yes	3 (0.5%)	0 (0)			
No	584 (99.5%)	107 (100%)			
Differentiation			0.823		
Well	161 (27.4%)	28 (26.1%)			
Medium	372 (63.4%)	66 (61.7%)			
Poorly	35 (6.0%)	8 (7.5%)			
Undifferentiation	19 (3.2%)	5 (4.7%)			
Depth of tumor invasion			< 0.001		
1–2	47 (8.0%)	25 (23.4%)		Reference	
3–4	540 (92.0%)	82 (76.6%)		0.281 (0.159–0.498)	< 0.001
Distance of tumor from the anal			0.178		
< 5	249 (42.4%)	55 (51.4%)			
5 ≤ X < 10	293 (49.9%)	47 (43.9%)			
≥ 10	45 (7.7%)	5 (4.7%)			
Tumor location			0.201		
Upper	34 (5.8%)	3 (2.8%)			
Middle	287 (48.9%)	47 (43.9%)			
Lower	266 (45.3%)	57 (53.3%)			
Tumor size by MRI			< 0.001		
< 2.5	168 (28.6%)	49 (45.8%)			
≥ 2.5	419 (71.4%)	58 (54.2%)			
Recurrent tumor			0.629		
Yes	6 (1.0%)	0 (0)			
No	581 (99.0%)	107 (100%)			
Tumor size by colonoscopy			0.006		
≤ 0.5	328 (55.9%)	75 (70.1%)			
> 0.5	259 (44.1%)	32 (29.9%)			
Initial CA125			0.279		
≤ 7.5	213 (36.3%)	33 (30.8%)			
> 7.5	374 (63.7%)	74 (69.2%)			
Initial CEA			0.011		
≤ 2.0	145 (24.7%)	39 (36.4%)			
> 2.0	440 (75.3%)	68 (63.6%)			
Initial CA199			0.053		
≤ 12.5	342 (58.3%)	73 (68.2%)			
> 12.5	244 (41.7%)	34 (31.8%)			
Initial CA153			0.945		
≤ 6.0	183 (31.2%)	33 (30.8%)			
> 6.0	404 (68.8%)	74 (69.2%)			
Initial AFP			0.318		
≤ 5.0	529 (90.1%)	93 (86.9%)			
> 5.0	58 (9.9%)	14 (13.1%)			
Preoperative CA125			0.023		
≤ 7.5	160 (27.3%)	18 (16.8%)		Reference	
> 7.5	427 (72.7%)	89 (83.2%)		0.425 (0.243–0.745)	0.003
Preoperative CEA			0.002		
≤ 2.0	200 (34.1%)	53 (49.5%)		Reference	
> 2.0	387 (65.9%)	54 (50.5%)		0.591 (0.380–0.920)	0.02
Preoperative CA199			0.011		
≤ 12.5	394 (67.1%)	85 (79.4%)		Reference	
> 12.5	193 (32.9%)	22 (20.6%)		0.519 (0.307–0.877)	0.014
Preoperative CA153			0.12		
≤ 6.0	75 (12.8%)	8 (7.5%)			
> 6.0	512 (87.2%)	99 (92.5%)			
Preoperative AFP			0.438		
≤ 5.0	446 (76.0%)	85 (79.4%)			
> 5.0	141 (24.0%)	22 (20.6%)			
CEA difference			0.251		
≤ 0	432 (73.6%)	73 (68.2%)			
> 0	155 (26.4%)	34 (31.8%)			
Ratio of CEA difference			0.251		
≤ 0	432 (73.6%)	73 (68.2%)			
> 0	155 (26.4%)	34 (31.8%)			
CA199 difference			0.436		
≤ 0	385 (65.6%)	66 (61.7%)			
> 0	202 (34.4%)	41 (38.3%)			
Ratio of CA199 difference			0.436		
≤ 0	385 (65.6%)	66 (61.7%)			
> 0	202 (34.4%)	41 (38.3%)			
Neoadjuvant radiotherapy			< 0.0001		
Yes	280 (47.7%)	78 (72.9%)		Reference	
No	307 (52.3%)	29 (27.1%)		0.356 (0.222–0.571)	< 0.001
Chemotherapy			0.687		
Single-agent	102 (17.4%)	17 (15.9%)			
Double-agent	387 (65.9%)	75 (70.1%)			
Triple-agent	98 (16.7%)	15 (14.0%)			

*LARC*, locally advanced rectal cancer; *BMI*, body mass index; *pCR*, pathologic complete response; *CA199*, carbohydrate antigen 199; *CA125*, carbohydrate antigen 125; *AFP*, alpha-fetoprotein; *CEA*, carcinoembryonic antigen

### Comparison between ML classifier and nomogram model

As shown in Table 3, we compared the performance of the aforementioned models. Undoubtedly, the ML classifier presented full range superiority in AUROC, sensitivity, and specificity over the nomogram model.

**Table 3:** Model performance for predicting pCR

Outcome	ML classifier		Nomogram model	
	Training set	Tuning set	Training set	Tuning set
AUROC	0.95	0.73	0.72	0.69
Sensitivity	82.2%	71.9%	43.0%	55.0%
Specificity	91.6%	70.0%	87.1%	78.5%

*pCR*, pathologic complete response; *ML*, machine learning; *AUROC*, area under the receiver operating characteristic curve

## Discussion

The introduction of standard management in LARC (NAT + TME) has improved cancer survival and local control in recent years. However, the physical, mental, and emotional burden caused by invasive surgery has opened the discussion on the trade-off between recurrence risk and better QOL in cCR patients. Given the evidence that the conservative approach, i.e., W&W, taken by cCR patients to avoid morbidity and functional sequelae resection was not inferior to surgery in terms of prognosis, W&W was reported as a safe alternative to major surgery [6, 8]. A questionnaire survey of patients’ perspectives showed that even by emphasizing the detailed risks toward LARC patients with cCR, 83% of them were willing to consider the W&W strategy, 94% of them were willing to bear the 25% recurrence rate, and 95% accepted intensive surveillance despite its being troublesome [31]. However, there were no uniform and reproducible criteria for cCR, which led to a low concordance rate between cCR and pCR. Previous studies have shown that the true pCR rate in those who were assessed as cCR ranged from 44 to 78%, and a proportion of approximately 10% pCR was misjudged as non-cCR. In addition, the sensitivity of biopsies was 12.9%, which was slightly consistent with 30.4% of the surgical specimens [32, 33]. Moreover, current restaging methods such as digital rectal examination, endoscopy, computed tomography, MRI, or positron emission tomography–computed tomography were not capable of being surrogate methods of surgical pathological assessment to observe tumor response. Therefore, a precise and objective tool for the preoperative assessment of pCR is urgently needed. To address this, we constructed an ML classifier to predict pCR, which demonstrated high performance, and compared it with the conventional linear model to realize individualized precision therapy.

The AUROC of our ML classifier in discriminating binary tumor response of LARC patients after NAT was 0.95, with a sensitivity of 82.2% and specificity of 91.6%, and it achieved slightly lower values in the tuning set with an AUROC of 0.73, sensitivity of 71.93%, and specificity of 70%. When we analyzed the dataset, we found that demographic characteristics, depth tumor invasion, preoperative CEA, neoadjuvant radiotherapy, CEA difference, and the ratio of CEA difference were not comparable between the training and tuning sets; whereas, it ranked the top few important parameters in both the ML classifier and nomogram model. This may fully explain the moderate performance on the tuning set. From the perspective of data analysis, the performance of our model could be optimized if we reassigned the training and tuning sets or performed propensity-score matching to ensure comparability of key features; however, our current results reflect daily clinical practice where protocols are subject to variations between different centers. Therefore, this may render our model more generally applicable. The tuning set conditions could exist in clinical practice, and we could not expect data distribution to meet the required standard. This, in another way, makes our model more representative even in some extreme situations. Based on these findings, we believe that the current results of the tuning set were the worst-case scenario that may prevail in clinical practice. However, the predictive ability was still far superior to that of the conventional linear regression model (nomogram). In addition, our ML classifier cannot only identify independent associated factors like that of the nomogram, but allows the determination of the internal law of the data for better use of the additional information than the linear model.

Neoadjuvant radiotherapy, which was recommended in various guidelines as part of the standard treatment in LARC patients, ranked first and second in feature importance in the ML classifier and nomogram, respectively. The results provide strong evidence for the guidelines once again. Previous studies have investigated the role of tumor size and pretreatment T stage as predictors of tumor response in LARC patients after NAT, and our study was consistent with their findings to show that indicators measured by MRI may help predict pCR outcomes [10, 34–36]. The use of CEA, CA125, and CA199 alone or in combination was reported to help in making decisions for digestive tract tumors or adenocarcinomas as prognostic indicators or in monitoring therapeutic effects [37–41], while the use of serological indications alone is insufficient for both sensitivity and specificity. These were in line with our findings; moreover, we found that indicator changes (e.g., CEA difference) before and after treatment may reflect treatment responses within the tumor to some extent. However, little research focused on this aspect, and this insight suggested a new view possibility in the monitoring of treatment response. Further studies are needed to investigate the value of indicator changes presenting in blood, radiology, or other areas.

Many studies predict pCR in LARC patients after NAT. van der Sande et al. [42] initiated a study in which three endoscopists predicted pCR using endoscopic features. The results showed that the sensitivity, specificity, and AUROC of human recognition fluctuated between 72 and 94%, 61 and 85%, and 0.80 and 0.84, respectively. The interobserver consistency was mild to moderate. The performance of our model was better than that of van der Sande et al. and a ML model could eliminate the empirical dependence of human recognition. Besides, Bulens et al. [43] constructed multiple MRI radiomics by T1, T2, and DWI sequences, in which the highest AUROC was 0.86. Also, Jin et al. [44] proposed a multi-task deep learning model to predict pCR using MRI imaging, the AUROC reached 0.97 when they combined imaging analysis with CEA, and the model was well externally validated in multi-center data. Although their model showed outstanding performance using artificial intelligence algorithm, different magnetic field intensity hampered them from clinical practice. However, our ML model was constructed using common clinicopathological parameters, which made it easy to apply to other centers.

This study has several limitations that restrict the interpretations of our findings. First, selection bias cannot be excluded because of the retrospective nature of this study. Second, our models were internally validated using a tuning set within our institution; thus, external validation is needed. Although pooling of data permits additional analyses, interinstitutional biases and variations in treatment practices may affect data interpretation. A large single-institution experience with standardized treatment and pathologic evaluation can avoid this limitation. To the best of our knowledge, this study included the largest number of cases in related research. Huang et al. [36] built several ML classifiers to find an artificial neural network (ANN) algorithm with the best performance in predicting tumor response compared with *k*-nearest neighbor, support vector machine, naïve Bayes classifier, and multiple logistic regression. The performance of the ANN model was similar to that of our XGBoost classifier, but the relatively small number of patients from the same cohort may impart substantial bias and poor generalizability. Finally, some variables included in our model were highly subjective and relied on personal experience. Despite these, we are making efforts to include more variables and patients to improve the predictive performance of the XGBoost model and collecting multicenter data for external validation. Once the predictive performance of the model meets the clinical needs, we will translate it into an online risk calculator that is free to the public [45]. Clinicians need to enter the specific values of the variables, and the calculator outputs the probability of pCR.

We constructed an ML classifier with a high-volume database using easily obtainable preoperative clinicopathological parameters to accurately predict the binary tumor response of LARC patients after NAT, which outperformed the conventional linear model in predicting ability. It may serve as a robust tool for tumor response prediction and improve QOL in patients with pCR due to the omission of major surgery.

## Supplementary Information

Below is the link to the electronic supplementary material.

Supplementary file1 (R 3 KB)
