# Correction to: Predicting pathologic complete response in locally advanced rectal cancer patients after neoadjuvant therapy: a machine learning model using XGBoost

**DOI:** 10.1007/s00384-022-04215-6

**Published:** 2022-07-14

**Authors:** Xijie Chen, Wenhui Wang, Junguo Chen, Liang Xu, Xiaosheng He, Ping Lan, Jiancong Hu, Lei Lian

**Affiliations:** 1 Guangdong Provincial Key Laboratory of Colorectal and Pelvic Floor Diseases, Guangdong Institute of Gastroenterology, The Sixth Affiliated Hospital, Sun Yat-Sen University, Guangzhou, Guangdong China; 2 Department of Gastrointestinal Surgery, The Sixth Affiliated Hospital, Sun Yat-Sen University, Guangzhou, Guangdong China; 3 Information and Data Centre, Guangzhou First People’s Hospital, School of Medicine, South China University of Technology, Guangdong, Guangdong China; 4 Department of Colorectal Surgery, The Sixth Affiliated Hospital, Sun Yat-Sen University, Guangzhou, Guangdong China; 5 Department of Pathology, The Sixth Affiliated Hospital, Sun Yat-Sen University, Guangzhou, Guangdong China; 6 Department of Endoscopic Surgery, The Sixth Affiliated Hospital, Sun Yat-Sen University, Guangzhou, Guangdong China; 7 Department of Network Management, The Sixth Affiliated Hospital, Sun Yat-Sen University, Guangzhou, Guangdong China

## Correction to: International Journal of Colorectal Disease (2022) 37:1621–1634 https://doi.org/10.1007/s00384-022-04157-z

In the original version of the above article, the equal contribution note was missing. The note should state:

Xijie Chen, Wenhui Wang, Junguo Chen and Liang Xu contributed equally to this article.

The original article has been corrected.

